# Superior Electrochemical Sensor Application of Co_3_O_4_/C Heterostructure in Rapid Analysis of Anticancer Drug Palbociclib in Pharmaceutical Formulations and Biological Fluids

**DOI:** 10.1021/acs.langmuir.4c02551

**Published:** 2024-09-28

**Authors:** Ozgul Vural, Nesrin Buğday, Asena Ayşe Genc, Nevin Erk, Ozgur Duygulu, Sedat Yaşar

**Affiliations:** † Faculty of Pharmacy, Department of Analytical Chemistry, Ankara University, 06560 Ankara, Turkey; ‡ The Graduate School of the Health Sciences, Ankara University, 06110 Ankara, Turkey; § Faculty of Science and Art, Department of Chemistry, İnönü Üniversity, 44280 Malatya, Turkey; ∥ TUBITAK Marmara Research Center, Materials Technologies, 41470 Gebze, Kocaeli, Turkey

## Abstract

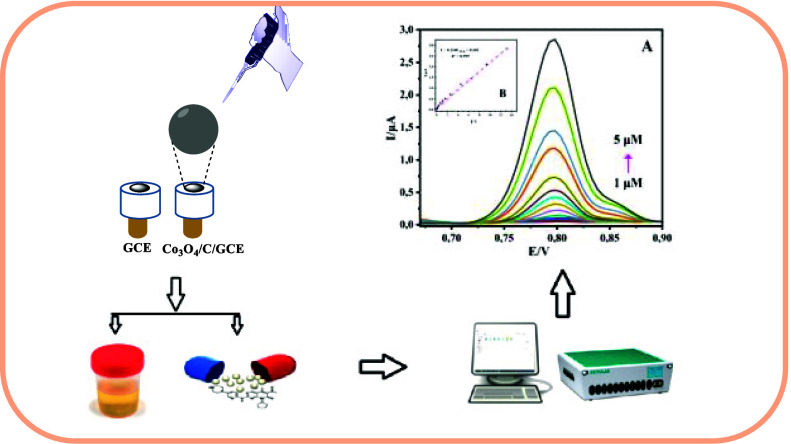

In this work, we report a study examining how different salt concentrations affect the structure and electrochemical performance of two Co_3_O_4_/C materials designed for the fabrication of an easy, cheap, fast, safe, and useful electrochemical sensor for the detection of Palbociclib (PLB). Co_3_O_4_ nanoparticles were successfully created by encapsulating them in N-doped amorphous carbon matrices by using the molten salt-assisted approach. In this process, different amounts of potassium iodate and zeolitic imidazolate framework-12 (ZIF-12) were used, followed by pyrolysis at 800 °C. Optimum Co_3_O_4_ embedded porous carbon structures were obtained, and the composite with the highest electrochemical properties was modified to a glassy carbon electrode (GCE) surface for PLB detection. The linear response spanned from 1.0 to 5.0 μM, featuring a limit of detection (LOD) of 0.122 μM and a limit of quantification (LOQ) of 0.408 μM; the correlation coefficient was calculated as 0.995. The high sensitivity of the method in detecting PLB in pharmaceutical samples and human urine demonstrated its feasibility, with recovery percentages ranging from 99.3% to 101.3% and relative standard deviation (RSD) values of <3%. Therefore, this technique will make a significant contribution to monitoring and improving existing cancer treatment options.

## Introduction

Breast cancer (BC) is one type of cancer that affects 2.3 million people all over the world each year. It is estimated that there were more than 140 000 women suffering from metastatic breast cancer (mBC) in 2018, while by 2025, it is projected that more than 169 000 women will have mBC. In particular, future prognostications indicate 3 million new breast cancer cases annually. This might lead to at least 1 million deaths each year.^1^ In fact, although medical science is making strides with newer avenues of treatment, breast cancer is still the second most widespread cancer affecting women. Therefore, there is a need for synthesis and analysis of drugs for breast cancer.^2−4^ An example is the use of small-molecule proteins such as targeted therapy that plays a crucial role in many forms of cancer treatments. These drugs have low molecular weight, which enable them to reach specific locations in cancer cells, where they first act on protein molecules. On the other hand, these proteins play key roles in cell cycle advancement. However, the accelerated multiplication is due to overactivation of these proteins leading to uncontrolled cell cycle control.^5^ Palbociclib (PLB) is the first FDA-licensed CDK4/6 inhibitor currently used in a large number of breast cancer patients. This regard, PLB acts like a very selective inhibitor that causes growth and division of breast cancer cells to be suppressed in relation to certain proteins. The effectiveness of this focused therapeutic approach in halting the advancement of cancer is hopeful.^6^ PLB is defined as a pyridopyrimidine compound containing both secondary and tertiary amines in its structure, belonging to the cyclopentane group.^7^ PLB detection has been accomplished through several techniques, encompassing RP-HPLC,^8,9^ spectrofluorimetric methods,^10^ UPLC,^11^ LC-MS/MS.^12,13^ Nevertheless, such analytic techniques usually require very complicated preprocessing steps, extremely lengthy experimental procedures, and expensive instruments and hence are not practical in routine drug testing.

Unfortunately, these mentioned methods exhibited the disadvantages of time-consuming, expensive instruments, and complicated pretreatment. Compared to these methods, the electrochemical technique has attracted a great deal of interest, because of its high sensitivity, simple operation, fast response speed, low cost, and real-time detection in situ condition.^14−19^

Electrochemical analysis of PLB was studied using various electrodes, such as a mercury electrode,^20^ NH_2_-MWCNT/GCE,^21^ BPAC/NiFe_2_O_4_/MnCoFe-LDH/GCE,^22^ and P(HEMA-MAGA)@MIP/GCE.^23^

Utilizing materials that catalyze the redox processes of analytes facilitates the modification of electrodes with such materials, thereby minimizing the influence of extraneous molecules and altering the oxidation potential. This approach simplifies electrochemical analysis. Incorporating conductive materials enhances the electrode performance, enabling the detection of electroactive compounds. Chemically modified composite electrodes reduce interference and allow for the sensitive detection of pharmaceuticals at very low concentrations.^24−26^

In comparison with traditional carbon paste electrodes (CPE), carbon composite electrodes using ionic liquids as binders show high conductivity, provision of fast electron transfer and antifouling properties, resulting in the improvement of voltammetric signal (overpotential decrease, decrease in the difference between peak potentials and peak current increase).^27−31^

However, for this method to be feasible, the analyte must possess certain structural characteristics.^32,33^ Specifically, they should contain functional groups readily oxidizable or reducible solely through the electric current generated during electrochemical measurements. Additionally, the analyte must exhibit a structure conducive to facile participation in redox processes.^34−37^

Analyte concentrations are typically low, necessitating enhanced electron transfer on unmodified electrodes to visualize the outcome of the electrochemical reaction.^38^ Moreover, the presence of multiple interfering molecules in complex matrices further complicates measurements, as these molecules may generate electrochemical signals within the same potential range as the target analyte, potentially causing selectivity issues.^39−42^ To mitigate interference from other molecules, electrode modification with materials capable of catalyzing the redox processes of analytes, altering their oxidation potential, proves advantageous.^24^ Additionally, improving electrode performance through the use of conductive materials enables the identification of electroactive compounds undergoing oxidation or reduction processes at the electrode surface, thereby facilitating electroanalytical investigations.^43−45^ Furthermore, chemically modified composite electrodes offer a valuable option for the selective identification of pharmaceuticals, ensuring proper separation of detection potentials and minimizing interference.^46−48^

Zeolitic imidazolate framework (ZIFs) has a wide variety of structural and chemical features.^49−52^ By undergoing pyrolysis at high temperatures or chemical reactions that can be controlled, metal–organic frameworks (MOFs) can be easily transformed to metal compounds that are embedded in porous carbon networks and possess distinctive compositions and architectures. The porous carbon materials produced by employing MOFs as precursors in this methodology find application in several domains due to their pore-rich structure, which facilitates complete exposure of the active sites and thus enhances the kinetics of electrochemical reactions in battery systems.^53,54^ Furthermore, heteroatom-rich carbon generated from MOFs has the capability to generate exterior flaws, hence enhancing the conductivity of the carbon matrix.^55,56^ Enhanced electrical conductivity, accelerated ion diffusion, and favorable structural stability of the electrode during the electrochemical process are all outcomes that can be achieved by utilizing the porous structure and N-rich carbon of the composite material.

Herein, we exploit carbon-coated Co_3_O_4_ composite structures with the external layer being a carbon coating derived from ZIF-12 by pyrolysis. Although these composite materials structurally contain the same components, they exhibit different electrochemical sensor performances due to different ratios of the components. This difference in electrochemical sensor performance is due to the concentration of the KIO_3_ salt used to synthesize the composite materials. Despite the pyrolysis process under argon, this method is ideal for the one-pot synthesis of metal oxides embedded in a porous carbon network. The composite structure of Co_3_O_4_/C samples provides materials with the following advantages. (1) The Co_3_O_4_/C structure creates an amorphous interface that enhances the rate of charge transfer. (2) Coating the carbon layer can contain the active material within the carbon shell, preventing fragmentation and agglomeration of active components and creating a seamless pathway for electron transport. Taking advantage of these benefits, Co_3_O_4_/C as the coating material for electrochemical sensor of PLB exhibits a high detect capacity of PLB, suggesting advantageous electrochemical capabilities. An electrochemical sensor material with enhanced performance characteristics is conceivably created through the implementation of a method that we have developed.

In the literature, there is no electrochemical method of PLB detection using the Co_3_O_4_/C/GCE type nanosensor. This study describes the electrochemical method developed using the Co_3_O_4_/C/GCE nanosensor for the detection of PLB. Thanks to the unique properties of this sensor, our research enabled the detection of PLBs with the Co_3_O_4_/C/GCE nanosensor with high sensitivity. A simpler and more beneficial option than analytically complex procedures is the modified electrode, which exhibits improvement in its selectivity as well as sensitiveness. Considering the high sensitivity and selectivity of the designed sensor based on the results obtained, this sensor can be effectively applied for accurate measurement of PLB. The nanosensor created with Co_3_O_4_/C composite may have more widespread use, due to its superior structural properties, compared to other modified electrodes used in the electrochemical analysis of PLB. These properties may also shed light on the identification of other anticancer drugs. Thus, this analytical study would be a potential way to advance the pharmaceutical field and the science.

## MATERIAL AND METHODS

### Reagents, Materials, and Apparatus

To be used in the studies, the following reagents were bought at Sigma–Aldrich Co. (Germany): 99.5% glucose; 98.0% l-arginine; l-methionine; sodium hydroxide; 99% K_3_Fe(CN)_6_; HCl; sodium acetate; ascorbic acid; 99.0% uric acid; acetic acid; potassium chloride; sodium phosphate; and sodium sulfate. These were analytical-grade chemicals that were used directly without further purity. The source for these reagents is Sigma–Aldrich Co., and more information can be found on the Sigma-Aldrich Co. Web site: https://www.sigmaaldrich.com (Germany). Phosphoric, boric, and acetic acids were dissolved in highly pure water to create a Britton–Robinson (B-R) buffer. The stock solution for PLB was prepared using a methanol:water at a 1:1 proportion. Chemical compounds were used directly, since they were of analytical purity.

XRD analysis of the prepared materials was performed using a Rigaku Rint 2000 X-ray Diffractometer between 2° 2θ/min and 80° 2θ/min with a scan rate of 2° 2θ/min. The specimens were investigated by using a JEOL Model JSM 6510LV scanning electron microscopy (SEM) system at 15 kV. The morphology at the nanoscale was further observed by a JEOL Model JEM 2100 high-resolution transmission electron microscope (LaB_6_ filament) operated at 200 kV and equipped with an Oxford Instruments X-Max 80T energy-dispersive spectrometry (EDS) system. Carbon support film coated copper TEM grids (Electron Microscopy Sciences, Catalog No. CF200-Cu, 200 mesh) were used. Images were taken by a Gatan Model 833 Orius SC200D CCD Camera. Gatan Microscopy Suite (GMS) 2 software was used. For diffraction pattern indexing, CrystBox software was used [M. Klinger, *CrysTBox—Crystallographic Toolbox*. Institute of Physics of the Czech Academy of Sciences, Prague, 2015. ISBN 978-80-905962-3-8. URL: http://www.fzu.cz/~klinger/crystbox.pdf]. The elemental composition and phase structure were analyzed by X-ray photoelectron spectroscopy (XPS) and were recorded using a Specs-Flex XPS instrument in the range of 200–4000 eV. Benzimidazole (BIM), Cobalt acetate tetrahydrate ((CH_3_COO)_2_Co·4H_2_O), sodium chloride (NaCl), potassium iodate (KIO_3_) and NH_4_OH (NH_3_, 28%–30% aqueous solution) were obtained from Sigma–Aldrich and used without any purification.

The electrochemical evaluation was conducted by utilizing the METROHM-Autolab potentiostat/galvanostat apparatus (Model 663 VA, software version Nova 2.1.1). Ag/AgCl (3 M KCl) was used as the reference electrode, GCE and Co_3_O_4_/C/GCE as the working electrodes, and a Pt wire purchased from BASi was used as the counter electrode in a three-electrode setup. The urine sample used in the experiment was provided by a volunteer.

### Synthesis of ZIF-12

ZIF-12 was synthesized according to our prior research with some modifications.^57^ In order to produce a uniform solution (solution A), the standard method involves dissolving 3.2 g of Co(NO_3_)_2_·6H_2_O in 80 mL of methanol/toluene (3:1). After that, we prepared a second uniform solution (solution B) by mixing 3 g of benzimidazole and 1.5 mL of NH_4_OH with another 80 mL of methanol/toluene (3:1). Following the addition of solution A, we rapidly stirred solution B for a duration of 3 h. We carefully washed the final product using methanol several times, followed by centrifugation for recovery, and then subjected the material to a drying process at a temperature of 50 °C for a duration of 24 h.

### Preparation of Co_3_O_4_/C Heterostructures

We synthesized the Co_3_O_4_/C heterostructures with several modifications based on our previous study.^57^ In a typical synthesis, 1 g of as-prepared ZIF-12 was added into 5 g of supersaturated salt solution (NaCl/KIO_3_, 9:1 and 8:2 by weight) under vigorous stirring for over 24 h until the precursor was encased by the salt solution. In a water bath, the temperature was progressively raised to 90 °C until the salt underwent complete recrystallization. The final product was achieved by drying under vacuum at 50 °C overnight. The ZIF-12@NaCl/KIO_3_ powder was subsequently heated under Ar at 800 °C for 3 h at a rate of 2 °C min^–1^. After cooling down to room temperature the product was obtained by washing with deionized water, followed by filtrating and drying at 120 °C. The products were named as Co_3_O_4_/C-10 and Co_3_O_4_/C-20, where 10 and 20 represents the weight ratio of used KIO_3_ salt.

### Preparation of the Modified Glassy Carbon Electrode

To prepare the GCE, the bare electrode underwent polishing with alumina slurries and was then sonicated in a mixture of equal volumes of deionized water and ethanol for 5 min. The composite was dispersed in deionized water and subjected to ultrasonication for 1 h. Subsequently, the resulting Co_3_O_4_/C-_10_/GCE nanocomposite suspension was utilized for the surface modification of GCE. A 6.0 μL amount of the Co_3_O_4_/C-10/GCE (0.1 M) suspension was cast onto the GCE surface and subjected to drying for 20 min, utilizing an infrared heat lamp.^58^ All electrochemical investigations were conducted utilizing Co_3_O_4_/C-10/GCE as the working electrode, platinum rod as the counter electrode, and Ag/AgCl saturated solution as the reference electrode. The EIS and CV procedures were executed in a solution of [Fe^–^(CN)_6_]^3–/4–^ (5.0 mM) and KCl (0.1 M). CV measurements were examined within a potential range from −0.5 to 1.0 V at a scan rate of 50 mV/s, and EIS studies were conducted across a frequency range of 10 kHz to 0.1 Hz at a potential of 0.1 V. In addition, DPV with a modulation amplitude and time of 0.05 V and 0.01 s, and a step potential of 0.005 V, was employed for the determination of PLB in B-R buffer at pH 2.0.^59^

### Preparation of Human Urine and Formulations of Pharmaceutical Doses

The novel sensor in this study was used for the precise determination of PLB across human urine and pharmaceutical tablets. The real samples were prepared according to our previous procedure.^60^ Prior to conducting any analysis on the urine specimen, the liquid portion obtained after centrifugation was sieved through a PTFE syringe filter, featuring a pore diameter of 0.45 μm.^61^ Using the conventional addition method, Co_3_O_4_/C-10 suitability for biological sample quantification of PLB was determined. Four capsules, each containing 125 mg of PLB, were precisely weighed and then ground into a powder. Initially, an amount equivalent to 4.47 mg of PLB is transferred into 10 mL volumetric flasks where it is then diluted with water. A simple filtration technique was used to separate crude inert components, which has limited. The filtrate obtained was subjected to filtration using a PTFE syringe filter with a pore size of 0.45 μm. This was succeeded by dilution with a buffer solution and subsequent exposure to a 15 min sonication process.

## Results and Discussion

### Synthesis and Characterization of Co_3_O_4_/C Heterostructures

**Figure 1:**
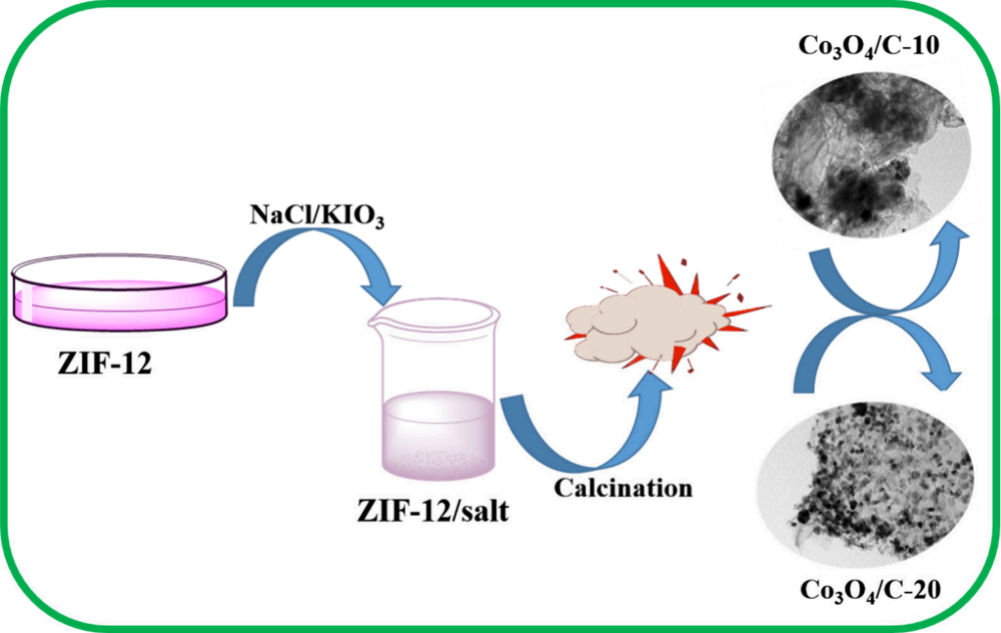
Synthesis procedures for Co_3_O_4_/C-10 and Co_3_O_4_/C-20 materials.

The synthesis process for the Co_3_O_4_/C-10 and Co_3_O_4_/C-20 materials is presented in Figure 1. The following is a description of the formation of the Co_3_O_4_/C-10 and Co_3_O_4_/C-20 materials: the mixture containing molten salts and ZIF-12 is calcined in an Ar atmosphere upon heating to ambient temperature.

**Figure 2:**
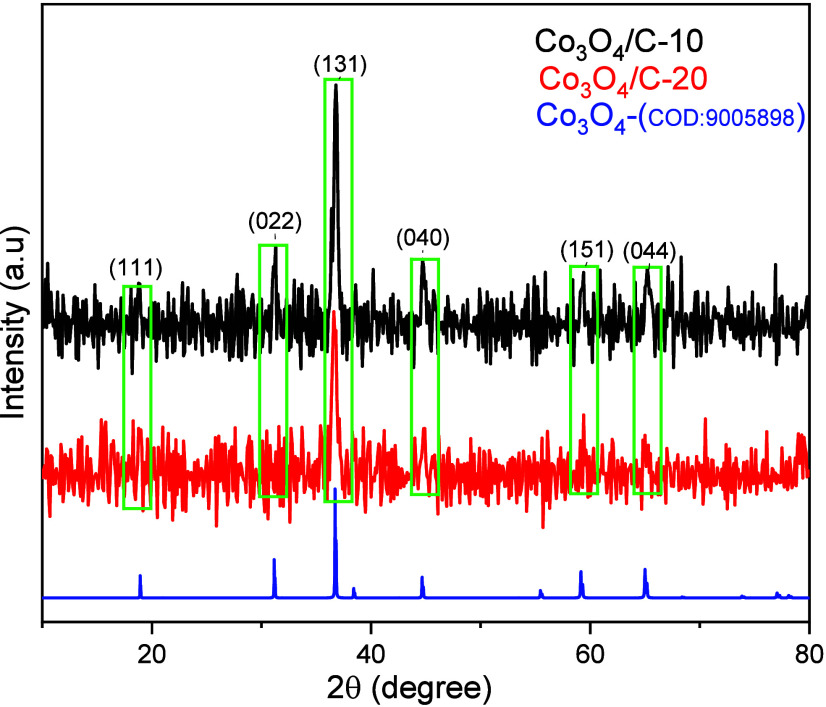
XRD analysis of Co_3_O_4_/C-10 and Co_3_O_4_/C-20.

**Figure 3:**
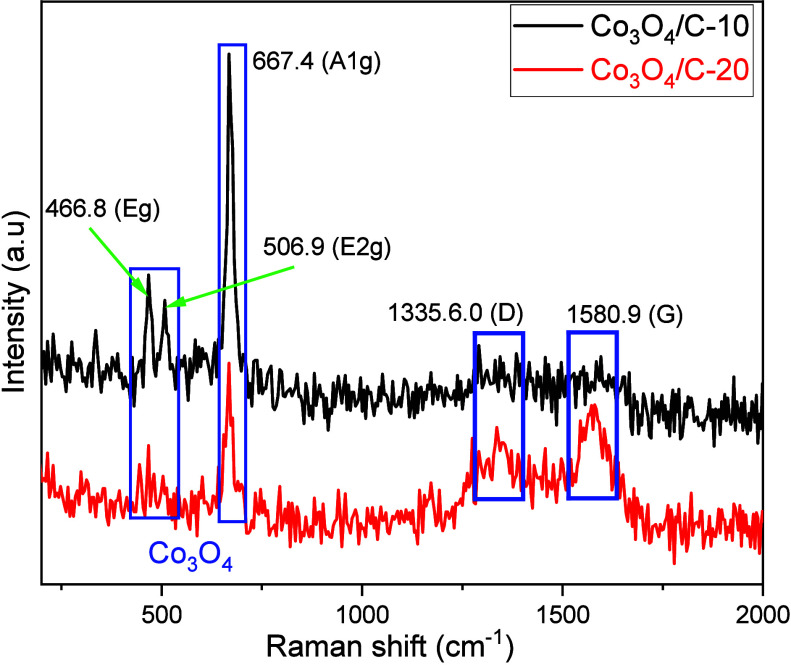
Raman spectra of Co_3_O_4_/C-10 and Co_3_O_4_/C-20.

The X-ray diffraction (XRD) patterns of the Co_3_O_4_/C-10 and Co_3_O_4_/C-20 materials are illustrated in Figure 2. According to XRD analysis of the Co_3_O_4_/C-10 and Co_3_O_4_/C-20, both materials mainly composed of amorphous carbon with Co_3_O_4_, which clearly reveals that the peaks of 18.86 (111), 31.03 (022), 36.57 (131), 44.46 (040), 58.88 (151), and 64.70 (044) well match with the JCPDS File No. 43-1003.^62^ There is no sharp peak at 26.6° in the XRD patterns of both materials; we think that the carbon structure is amorphous rather than graphite structure. This is also supported by the TEM analyzes. The above XRD analysis indicates that ZIF-12 containing cobalt in the molten salt medium have been transformed to mainly Co_3_O_4_ crystals by pyrolysis, resulting in the formation of the Co_3_O_4_/C materials. The structures of the Co_3_O_4_/C-10 and Co_3_O_4_/C-20 materials were elucidated by Raman analysis (Figure 3).

For Co_3_O_4_/C-10, the peaks at 466.8, 506.9, and 667.4 cm^–1^ correspond to E_g_, E_2g_, and A_1g_ modes of vibration, respectively.^63^ For Co_3_O_4_/C-20, the peaks at 466.8 and 506.9 were not observed as much as that of Co_3_O_4_/C-10 material. The vibration observed at 667.4 cm^–1^ in the case of Co_3_O_4_/C-20 sample belongs to the A_1g_ mode of vibration. The literature states that the Raman peaks of bulk Co_3_O_4_ are found at 482.4, 521.6, and 691 cm^–1^, respectively.^64^ The band at 667.4 cm^–1^ (A_1g_) for Co_3_O_4_/C-10 and Co_3_O_4_/C-20 represents the symmetric Co–O stretching vibration, classified as the A_1g_ species symmetry. The E_g_ and E_2g_ are vibrational modes that result from the combination of the tetrahedral site and octahedral oxygen in the Co_3_O_4_ system.^65^ The presence of Co_3_O_4_ in spinel structures is further confirmed by these peaks in both Co_3_O_4_/C-10 and Co_3_O_4_/C-20.^66^ The *I*_D_/*I*_G_ ratios for Co_3_O_4_/C-10 and Co_3_O_4_/C-20 were calculated as 1.12 and 1.33, respectively.

**Figure 4:**
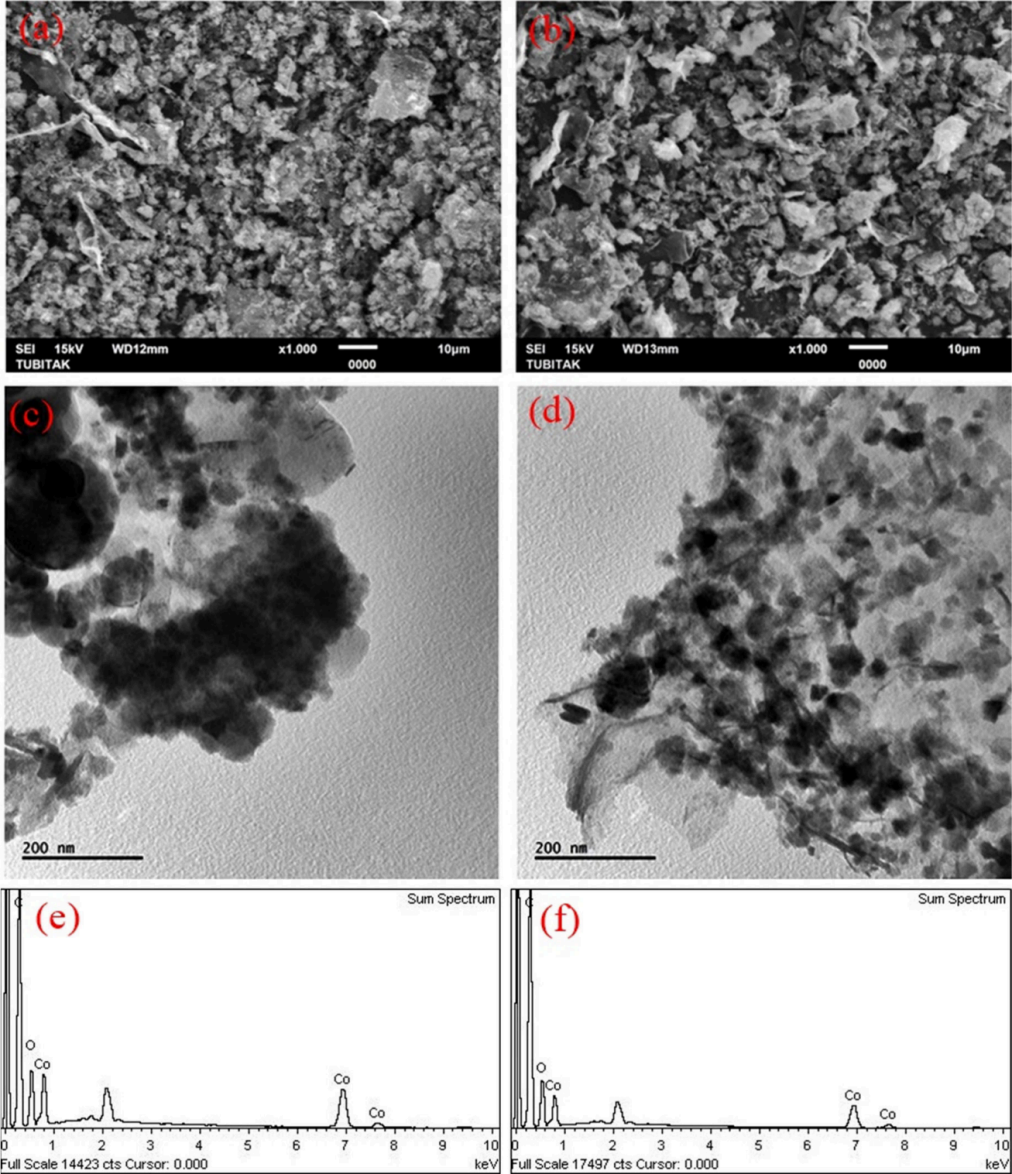
SEM, TEM, and SEM-EDS images of the (a, c, e) Co_3_O_4_/C-10 sample and (b, d, f) Co_3_O_4_/C-20 specimens.

The scanning electron microscopy (SEM), SEM-EDS, and SEM-EDS mapping analyses of Co_3_O_4_/C-10 and Co_3_O_4_/C-20 specimens are given in Figure 4 and Figure S2, respectively.

After the high-temperature carbonization process, the dodecahedron structure of ZIF-12 (Figure S3c) was completely dispersed and the size of the particles decreased. The both samples have been found to have 2D carbon structures. The Co_3_O_4_/C-10 sample has a more homogeneous morphology, with less aggregation than the Co_3_O_4_/C-20 sample. In Figure 4 and Figure S1, TEM images of Co_3_O_4_/C-10 and Co_3_O_4_/C-20 samples are shown, respectively. Thin amorphous carbon layer matrix of Co_3_O_4_/C-20 is more obvious in Figure 4d. It is also evident that the nanoparticle sizes of the two specimens are comparable. With the help of SEM-EDS it can be stated that the majority of the nanoparticles for both samples are Co_3_O_4_ (Figures 4e and 4f). The carbon and cobalt contents of the Co_3_O_4_/C-10 and Co_3_O_4_/C-20 materials can be assessed through SEM-EDS analysis. The carbon and cobalt content of the of the Co_3_O_4_/C-10 and Co_3_O_4_/C-20 were determined to be 53.16% and 31.01% and 58.32% and 23.40%, respectively, as shown in Table 1. The higher carbon and lower cobalt content of the Co_3_O_4_/C-20 can be related to the used higher concentration of KIO_3_ during the synthesis of material. Although we have no information about how this happened, it should be noted that the only difference between the synthesis of the two materials is the concentration of the KIO_3_ salt. This high carbon and lower cobalt content of Co_3_O_4_/C-20 can be directly related to the lower conductivity of Co_3_O_4_/C-20 than Co_3_O_4_/C-10 material.

**Table 1:** Elemental Composition and BET Surface Area of Co_3_O_4_/C-10 and Co_3_O_4_/C-20 Samples Obtained by SEM-EDS and N_2_-Adsorption Analysis

sample	C (%)	Co (%)	O (%)	BET surface area (m^2^/g)
ZIF-12	–	–		337
Co_3_O_4_/C-10	53.16	31.01	15.83	123.34
Co_3_O_4_/C-20	58.52	23.40	18.09	273.96

**Figure 5:**
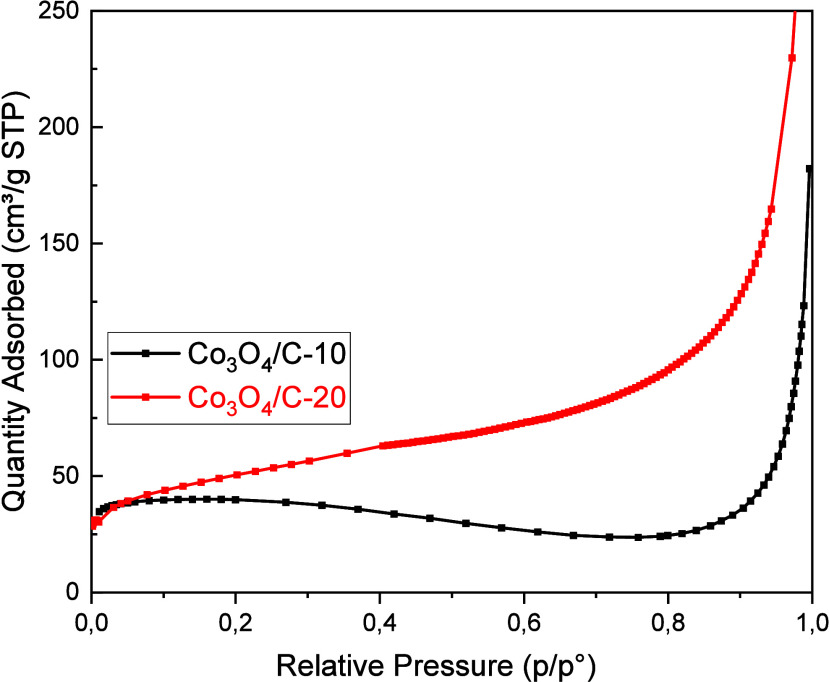
N_2_ adsorption isotherms of Co_3_O_4_/C-10 and Co_3_O_4_/C-20.

The nitrogen gas absorption curves and pore diameter distribution of Co_3_O_4_/C-10 and Co_3_O_4_/C-20 suggest that the concentration of the melting salt can greatly enhance the specific surface area of Co_3_O_4_/Cs (Figure 5). Table 1 lists the BET surface areas of the ZIF-12, Co_3_O_4_/C, Co_3_O_4_/C-10, and Co_3_O_4_/C-20 materials. The Co_3_O_4_/C-20 exhibited the highest surface area after ZIF-12, as indicated in Table 1. The structural collapse of ZIF-12 may have been caused lower surface area. The BET analyses confirmed that the Co_3_O_4_/C-10 and Co_3_O_4_/C-20 materials exhibited a microporous structure, in agreement with the observations from SEM and TEM images. Analysis of the adsorption diagrams shows that all samples exhibit Type 1 isotherms, demonstrating the presence of a microporous pore structure in Co_3_O_4_/C-10 and Co_3_O_4_/C-20.

**Figure 6:**
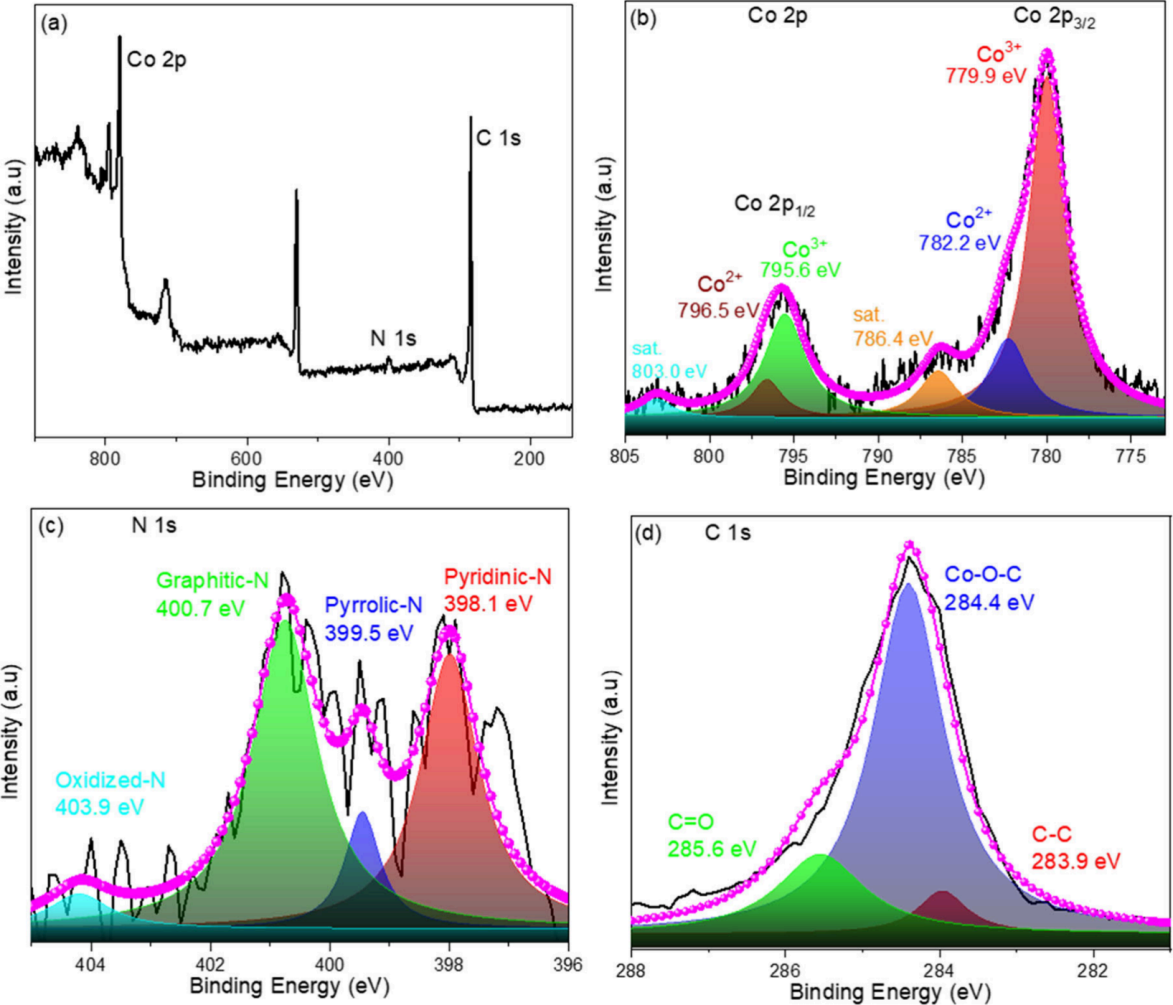
XPS spectra for (a) Co_3_O_4_/C-10 and high-resolution spectra of (b) Co 2p, (c) N 1s, and (d) C 1s.

Figure 6 displays the raw and adjusted XPS analysis data for Co 2p, C 1s, and N 1s in the Co_3_O_4_/C-10 material. The peaks for Co 2p, O 1s, N 1s, and C 1s are situated at 780.1, 530.3, 399.6, and 284.3 eV, respectively, as depicted in Figure 6a. The high-resolution Co 2p X-ray photoelectron spectroscopy (XPS) spectrum displays four primary peaks at 779.9, 782.2, 795.6, and 796.5 eV, along with two satellite peaks at 786.4 and 803.0 eV. Peaks at 779.9 and 782.2 eV correspond to Co 2p_1/2_, whereas peaks at 795.6 and 796.5 eV correspond to Co 2p_3/2_ of Co_3_O_4_, indicating the presence of Co^2+^ and Co^3+^ oxidation states.^67^ The XPS analysis indicates that the carbon concentration in Co_3_O_4_/C-10 is ∼70.15 at. %. The peaks at 283.9, 284.4, and 285.6 eV correspond to C–C, Co–O–C, and C=O, respectively. The detailed N 1s spectra of Co_3_O_4_/C-10 illustrates the presence of pyridinic-N, pyrrolic-N, graphitic-N, and oxidized-N at 398.1 399.5, 400.7, and 403.9 eV, respectively. The pyridinic-N and graphitic-N groups in the sample enhance electron transport within the composite carbon network of Co_3_O_4_/C-10. The N element content in NPCs is ∼4.54 at. %, as determined by XPS analysis (Table S1).

### Electrochemical Behavior of the Sensing Platform

**Figure 7:**
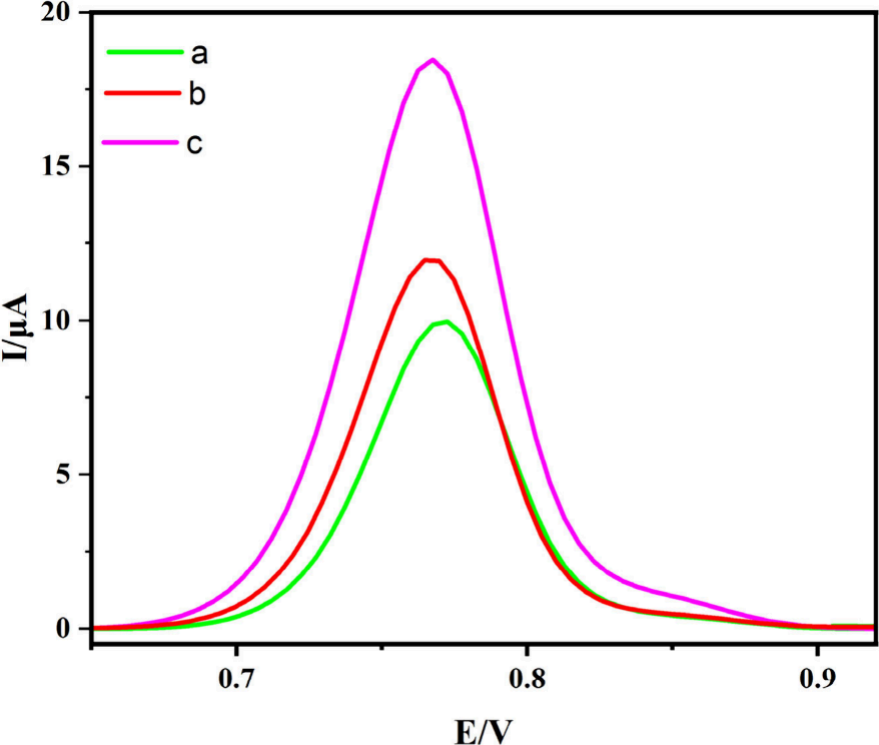
DPVs of (a) bare/GCE, (b) ZIF-12/GCE, and (c) Co_3_O_4_/C-10/GCE in 0.1 M BR solution (pH 2.0) containing 0.1 mM PLB.

Differential pulse voltammetry was used to assess the electrode activity of bare GCE, ZIF-12/GCE, and Co_3_O_4_/C-10/GCE in order to find 0.1 mM PLB in 0.1 M BR buffer and pH 2.0 (Figure 7). The anodic peak current of bare GCE was raised by adding 0.1 mM PLB; it displayed 9.97 uA at 0.773 V. This indicates that PLB oxidation occurs on the bare GCE surface. Using the Co_3_O_4_/C-10/GCE electrode increased the anodic peak current to 18.46 μA at 0.768 V; This increase is explained by the fact that the modified electrode outperformed the bare GCE in PLB detection.^68,69^ Therefore, considering that Co_3_O_4_/C-10/GCE has a higher conductivity than bare GCE and probably has multiple active sites on its surface, it can be explained that it has a higher conductivity upon its electrochemical oxidation. That is, it shows that there is a significant improvement (∼2-fold) in the electrochemical reactivity of PLB at the Co_3_O_4_/C-10/GCE electrode. This is evidenced by the increase in anodic peak current. Thus, it can be suggested that compared to bare GCE, the modified electrode has higher catalytic activity. Consequently, the electrochemistry properties of Co_3_O_4_/C-10/GCE demonstrate great prospects toward possible applications and advancement of electrochemical measuring technique.

**Figure 8:**
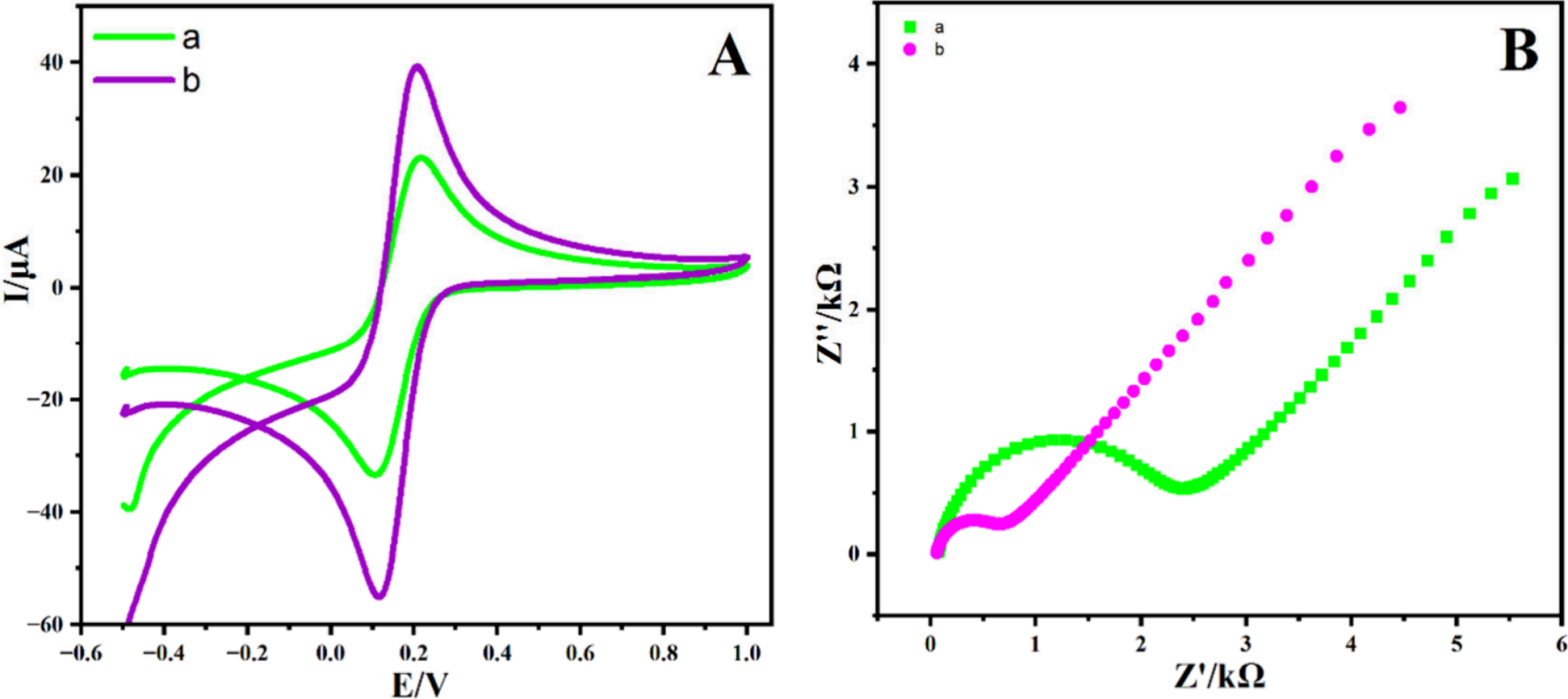
(B) EIS Nyquist plots and (A) CVs of the bare GCE (trace (a)), Co_3_O_4_/C-10/GCE (trace (b)), and Fe(CN)_6_]^3–/4–^ containing 0.1 mmol L^–1^ KCl were scanned at a rate of 50.0 mV s^–1^.

The electrochemical sensitivity of both bare GCE and Co_3_O_4_/C-10-modified GCE nanosensors was measured by using cyclic voltammetry (CV). Both nanosensor’s CV measurements were conducted at a concentration of 5.0 mM [Fe(CN)_6_]. As shown in Figure 8A, using Co_3_O_4_/C-10/GCE resulted in high oxidation peak current values. Unmodified electrode had oxidation peak potential value of Δ*E*_p_ = 102.50 mV, which compared to Co_3_O_4_/C-10/GCE of Δ*E*_p_ = 87 mV. These happen due to the role played by Co_3_O_4_/C-10 in providing electrocatalytic activity and increasing the electron transfer rate. The electrical conductivity of the electrodes formed from unmodified bare GCE and Co_3_O_4_/C-10-modified GCE was measured by EIS. Figure 8B, R_ct_ of the modified Co_3_O_4_/C-10/GCE showed an impedance value of 736.32 Ω, which far exceeded the impedance value obtained from the unmodified electrode (2369.40 Ω). By reducing the charge transfer resistance, modifying GCE with Co_3_O_4_/C-10 may improve the electron’s transmission kinetics at the electrode.

In order to generate a maximum number of microzones for electrochemical reactions, the electroactive surface area should be carefully looked into during the design process for electrode. Therefore, in order to ensure improved electrochemical quality, the electrode’s surface area must be expanded (Figures S4 and S5).

The electroactive surface areas (ESA) of the electrodes were determined by utilizing the slope of the cathodic peak current versus the square root of the scan rate and the Randles–Sevick (eq S1). The ESA of Co_3_O_4_/C-10/GCE was calculated to be 0.1906 cm^2^, surpassing that of the bare electrode (0.095 cm^2^). This finding suggests that the Co_3_O_4_/C-10-modified GCE is poised to exhibit superior electrocatalytic performance compared to the bare GCE. Thus, it may be reasoned that the enhanced conductivity and synergy might explain the rise in Co_3_O_4_/C-10/GCE’s surface area.

### Optimization of Important Factors

Optimization should be carried out with respect to the supporting electrolyte’s type of the modified electrode, its content, and amount. Initially, the best buffer solutions needed for use in this process are chosen. To this end, different buffers such as Britton–Robinson (BR), Phosphate-buffered saline (PBS), potassium chloride (KCl), acetate buffer (AC), NaOH, etc., were studied. PLB’s potential peak and oxidation current connection employing these electrolytes is depicted in Figure S6A. The choice to focus additional research on this particular electrolyte was made, since the BR buffer displayed the biggest current peak in comparison to the other buffers. The effect of different concentrations of Co_3_O_4_/C-10 composite solution on nanosensor performance was investigated. Concentrations ranging from 0.1 M to 2.0 M were tested, with the best performance achieved at 0.1 M. Higher concentrations led to increased layer thickness on the electrode surface, negatively affecting sensor performance and resulting in lower current values.^70^

**Figure 9:**
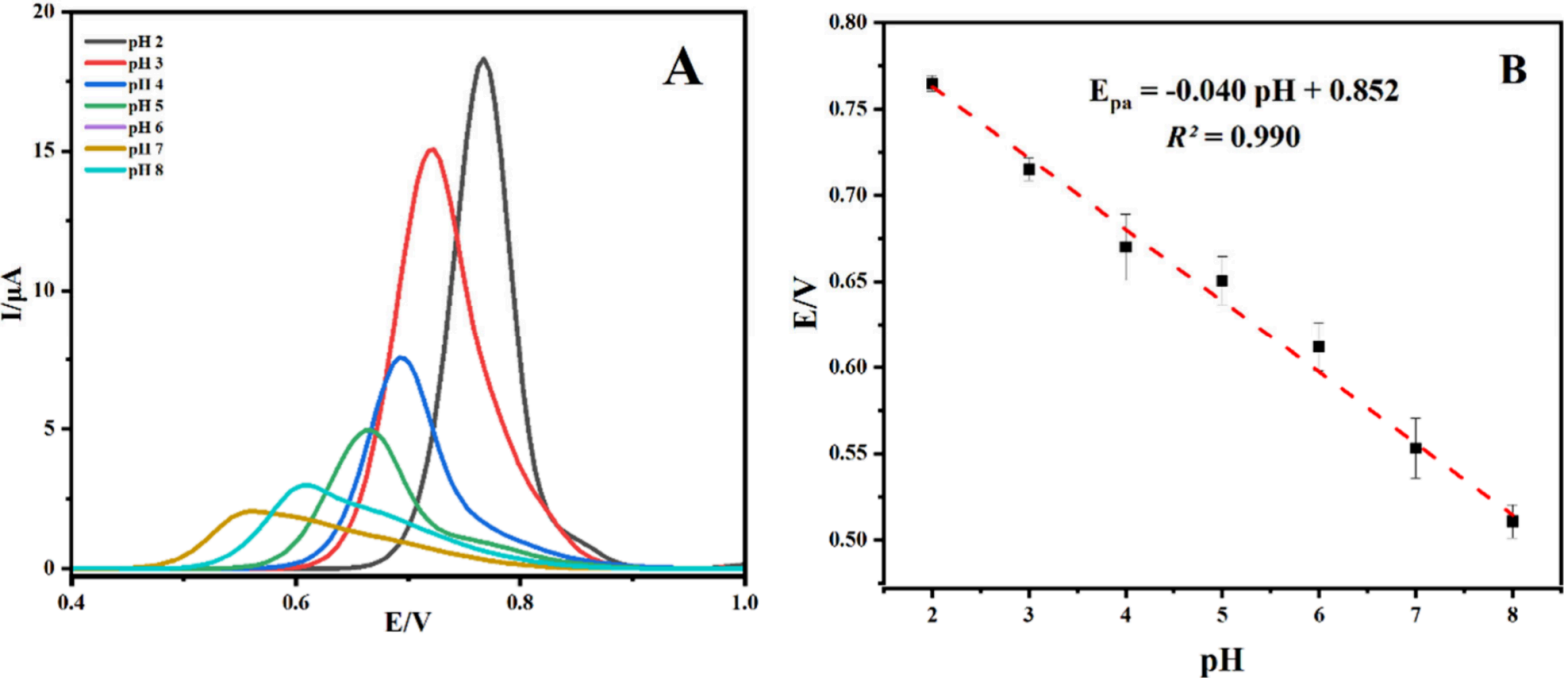
(A) DPV curves from Co_3_O_4_/C-10/GCE in BR and 0.1 mM PLB at various pH levels, as well as (B) the correlation between the pH value and peak potential (*E*_p_).

The range of 2.0–10.0 μL was assessed in order to determine the optimal amount of Co_3_O_4_/C-10 composite (Figure S6C). As seen in Figure S6C, the oxidation peak current of Co_3_O_4_/C-10 composite reached the maximum value of 6.0 μL. It was suggested that the decreases in oxidation peak current at concentrations above 6.0 μL may be due to adhesion failure between the modifier layer and the electrode surface. The Co_3_O_4_/C-10 composite concentrations 0.1 M and 6.0 μL gave the best conditions. It is through DPV that the electrochemical behavior of PLB was observed across a pH range of 2.0 to 8.0, using 0.1 M BR buffer (see Figure 9).

The pH value of the solution is one of the factors that can significantly affect the analyte signal. DPV voltammograms were recorded at different pH values, and a distinct peak was observed at pH 2.0. By elevation of the pH of the working solution, the corresponding oxidation peak current decreases, suggesting that protons actively participate in the mechanism of the PLB oxidation reaction. This indicates that protons are involved in the electrochemical oxidation process of PLB.^71−73^ The number of electrons in the PLB oxidation was estimated as equal.^74^ An important outcome of this investigation is the development of a linear regression model expressing the relationship between the oxidation peak potential of PLB and the surrounding pH levels as *E*_pa_ = −0.040pH + 0.852. This model, supported by a high coefficient of determination (*R*^2^ = 0.990), demonstrates exceptional accuracy in capturing the pH dependency inherent in the electro-oxidation of PLB. The key deduction from this complex electrochemical narrative is significant: both electrons and protons play an equitable role in the irreversible oxidation process of PLB at the Co_3_O_4_/C-10/GCE interface. This assertion is reinforced by the observed slope (40 mV/pH) of the relevant oxidation potential curve, which closely approaches the theoretically expected Nernstian slope value of 59.2 mV/pH.^75^

**Figure 10:**
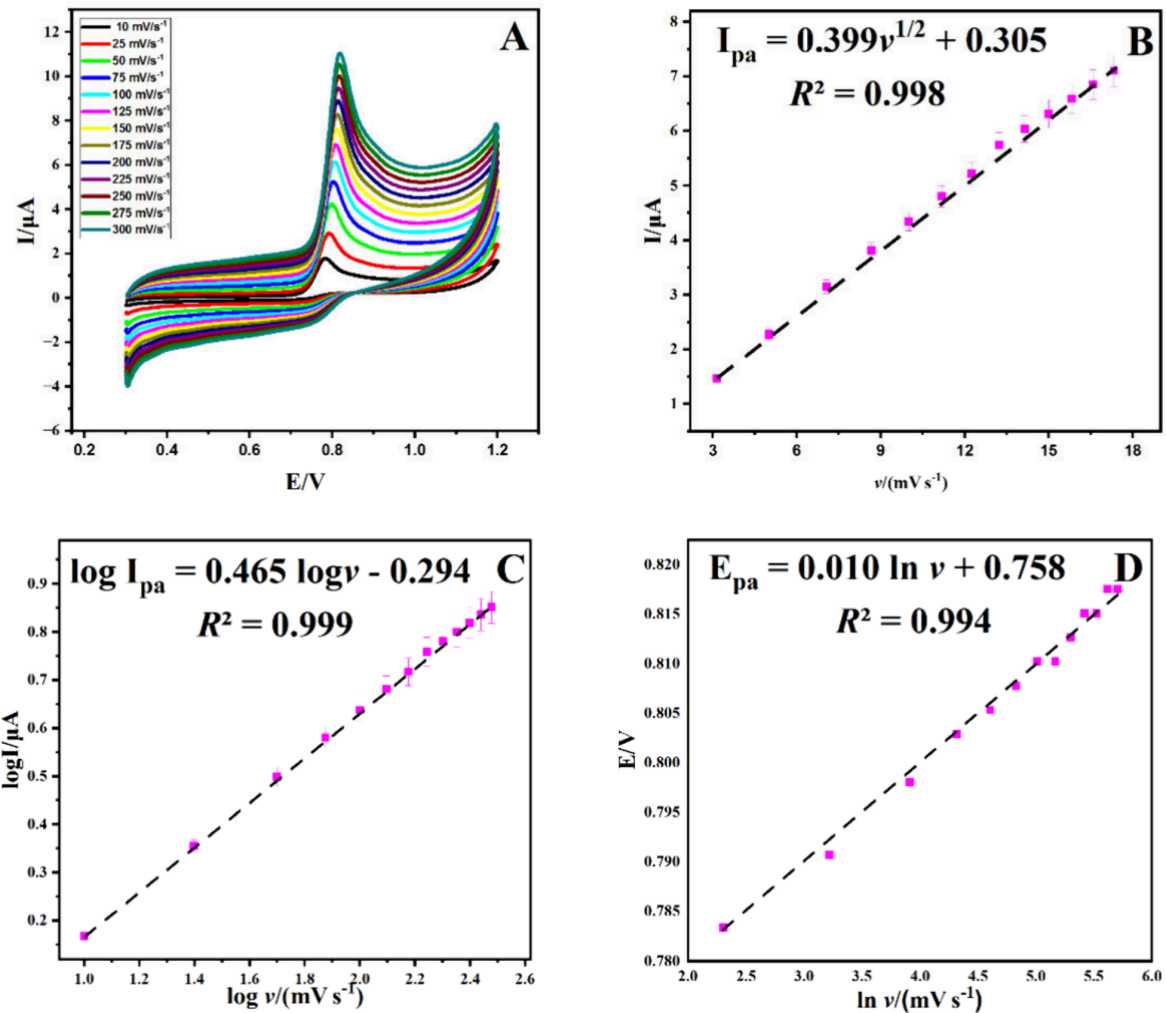
Plots of *I*_pa_ vs *v*_1/2_, log *I*_pa_ vs log *v*, and *E*_pa_ vs ln *v* are shown in panels (A) and (C) on the Co_3_O_4_/C-10/GCE, respectively, and at various scan speeds (10.0–300.0 mV s^–1^). (B) Linear plot of anodic peak currents versus square root of scan rates; (D) dependence of natural logarithmic of the scan rate on the peak potential *E*_p_/V.

The electro-oxidation properties of PLB were investigated by means of optimal conditions, cyclic voltammetry (CV) studies with the Co_3_O_4_/C-10/GCE electrode. Various scan speeds between 10 and 300 mV/s were used to determine the distinctive characteristics of PLB. It was discovered that the PLB’s oxidation peak moved in a positive direction, and the peak current rose when the scanning rate increased (see Figure 10A). Additionally, eq 1 was found, indicating a linear correlation (Figure 10B).
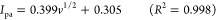
1

A logarithmic link was also found, as indicated by eq 2 (Figure 10C).
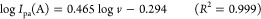
2

Figures 10B and 10C illustrate how the electrocatalytic oxidation of PLB is a diffusion-controlled process.^76^ The link between the maximum potential (*E*_pa_) in scanning speed and the logarithmic mean was also determined, as shown in eq 3.
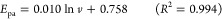
3

The Tafel plot (eq S2) was used to calculate the number of electrons transported, and the number of electrons was found to be ∼2.14 (Figure S7). Our study is consistent with the work of Alghamdi et al., who showed that the electrochemical behavior of PLB is probably from the electrochemical reduction of the peripheral carbonyl group (located outside the rings) to the hydroxyl alcohol group (eq 4). The suggested mechanism for the electrochemical reduction of PLB compounds is present in eq 4. This may contribute to the understanding of the electrochemical properties of PLB and may be important for drug development or analytical applications in a broader context.
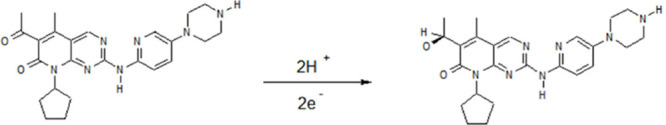
4

### Analytical Approach

**Figure 11:**
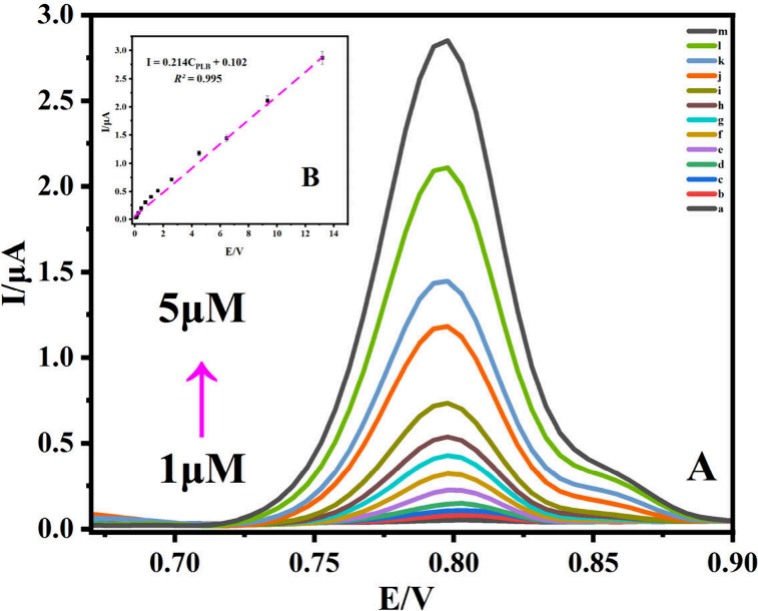
PLB’s DPV at various concentrations: (a) 1.0 μM, (b) 1.5 μM, (c) 2.0 μM, (d) 2.1 μM, (e) 2.3 μM, (f) 2.6 μM, (g) 3.0 μM, (h) 3.5 μM, (i) 3.6 μM, (j) 3.8 μM, (k) 4.0 μM, (l) 4.5 μM, (m) 5.0 μM) and in B–R buffer (pH 2.0) is shown in panel (A), and the plot of *I*_pa_ against PLB concentration is shown in panel (B).

The results of examinations of PLB solutions at various concentrations on the Co_3_O_4_/C-10/GCE electrode surface at optimal pH and temperature settings are shown in Figure 11.
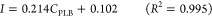
5

Equation 5 is the applicable linear equation. For the PLB oxidation peak currents and concentrations, which ranged from 1.0 to 5.0 μM, the results showed positive linearity. The limits of detection (LOD) and limits of quantification (LOQ) were carefully determined using eqs 6. and 7, respectively.^75^
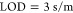
6
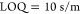
7

These formulations provide a detailed assessment of the analyte’s minimum detectable and quantifiable concentrations. In our study, the LOD and LOQ were found to be 0.122 and 0.408 μM, respectively (Table 2). These findings highlight the significant sensitivity and accuracy of the designed sensor, emphasizing its capability to detect PLB at very low concentrations. This characteristic is crucial for enhancing the sensor’s applicability in pharmaceutical analysis and clinical settings. The analytical performance of Co_3_O_4_/C-10/GCE was evaluated against previously reported PLB electrochemical sensors, with the results summarized in Table 2.

**Table 2:** Comparison of Electrochemical Sensors for the PLB

detection principle	electrochemical reduction	electrochemical oxidation	electrochemical oxidation	electrochemical oxidation	electrochemical oxidation
electrode	mercury electrode	NH_2_-MWCNT/GCE	BPAC/NiFe_2_O_4_/MnCoFe-LDH/GCE	ACR-PLB-MIP/GCE	Co_3_O_4_/C-20/GCE
voltammetric technique	SWV	DPV	DPV	DPV	DPV
linear range	0.1–1 μM	0.2–2 μM	0.01–13.0 μM	2.5 × 10^–11^ −2.5 × 10^–10^ standard solution (tablet)	1–5 μM
				2.5 × 10^–11^ – 2.5 × 10^–10^ commercial human serum sample	
LOD	8.8 × 10^–11^ M	4.82 × 10^–8^ M	3.5 × 10^–9^ M	1.35 × 10^–12^ standard solution (tablet)	0.122 × 10^–6^ M
				3.33 × 10^–12^ commercial human serum sample	
Repeatability	0.03%	2.37%	1.30%	0.42% standard solution	2.8%
				0.89 commercial human serum sample	
Recovery rate	93% and 86.4%	99.90%	100.9% and 100.4%	101.56 standard solution	99.3% and 101.3%
				101.06–98.67 commercial human serum sample	
Sample analysis	human urine and plasma	tablet	human urine and tablet	tablet and commercial human serum	human urine and tablet
ref	(20)	(21)	(22)	(23)	this work

### Reproducibility, Repeatability, Potential Interferents, and Stability of Co_3_O_4_/C-10/GCE

Nine consecutive measurements of DPV were performed in 0.01 mM PLB solution using Co_3_O_4_/C-10/GCE are shown in Figure S8A; the RSD was found to be 2.20%, indicating the repeatability of the developed sensor (Figure S8A). Moreover, 10 different electrodes were fabricated following the same technique to verify the reproducibility of the sensor (Figure S8B). The obtained RSD value was 2.8%, thus proving that the modified electrode is reliable. Therefore, the electrochemical sensor should be preferable toward having a selective response toward the target analyte in comparison with other possible interferents. As a result, a key element in assessing the process’s correctness is analytical selectivity.

Interfering substances commonly found in real and pharmaceutical samples were introduced into the electrochemical cell. These substances included ascorbic acid, uric acid, d-glucose, l-arginine, l-methionine, potassium chloride, sodium sulfate, potassium nitrate, and dopamine. Their concentrations were maintained at a level 100 times higher than that of the target analytes (PLB). The RSD values obtained were ≤2.00% (Table S2). The results indicated that these concentrations of ascorbic acid, uric acid, d-glucose, l-arginine, l-methionine, potassium chloride, sodium sulfate, potassium nitrate, and dopamine did not significantly affect the oxidation peak current of PLB at the Co_3_O_4_/C-10/GCE surface.^77^ These findings suggest that the interfering substances did not impact the performance of the Co_3_O_4_/C-10/GCE sensor in the analysis of the PLB.

For stability testing, the electrode was stored at room temperature in the laboratory, and measurements were repeated over 5 days (Figure S8d). After 5 days, the electrode retained 96.33% of its initial response. These results indicate that Co_3_O_4_/C-10/GCE has good stability and can be used to detect the PLB.

### Analysis of Real Samples

For the validation of the suitability of the analytic method with the developed electrode, the target analyte was determined in human urine samples and pharmaceutical samples (i.e., tablets). The recovery values, which range from 99.3% to 101.3%, show remarkable accuracy and are shown in Table 3. From this, we can state that Co_3_O_4_/C-10/GCE is a true selective electrochemical sensor for the direct detection of PLB in biological as well as pharmaceutical samples. These recovery values present firm proof toward the success and sensitivity observed from the analysis, thus verifying the credibility of this electrochemical sensor for genuine sample examination.

**Table 3:** PLB Determination Was Performed Using Actual Samples

added (μM)	found (μM)^a^	recovery (%)	RSD (%)
**Sample: Human Urine**			
0.4	0.405	101.3	0.5
0.6	0.603	100.5	1.8
0.8	0.807	100.9	2.6
**Sample: Tablet**			
0.4	0.403	100.8	0.9
0.6	0.608	101.3	1.4
0.8	0.794	99.3	0.1

a Three duplicate measurements were used to calculate the mean value.

In order to evaluate the intraday and interday accuracy and precision of the developed method, three different concentrations of PLB solution (4.0, 6.0, and 8.0 μg/mL) were tested under selected conditions. Analyses were performed on the same day (intraday) and on five different days (interday) within 1 week. Three separate analyses were performed for each concentration. The concentration was found by substituting the peak current values in the regression equation obtained for the standard PLB solution. Accuracy was given as the percent recovery values of the concentrations found, while precision was expressed as the relative standard deviation (%RSD) from the determined concentrations. The mean intraday and inter day recovery values between 100.47% and 100.6% showed that the accuracy of the method was excellent. The mean relative standard deviation (%RSD) values of the experiments performed intraday and interday were found to be between 0.10 and 1.40% and 0.66–1.41%, respectively (Table 4).

**Table 4:** Interday and Intraday and Accuracy and Precision of PLB Determination by DPV Method (*n* = 5)

	Intraday		Interday	
concentration (μg/mL)	recovery^a^ (%)	%RSD^b^	recovery^a^ (%)	%RSD^b^
4.00	100.80	0.90	101.27	0.66
6.00	101.30	1.40	100.73	1.41
8.00	99.30	0.10	99.81	0.86
				
mean	100.47	0.8	100.6	0.98

a Mean of three determinations (*n* = 5).

b RSD is relative standard deviation.

## Conclusions

Significant advancements have been made in electrochemical nanosensors and sensing over the past decade. This growing interest is related to their portability, low sample volume requirements, fast response times, and absence of the need for additional separation steps. As a result, a new, highly effective, rapid, and user-friendly electrochemical method has been developed for evaluating the PLB in pharmaceutical samples and human urine. The novelty of this study lies in the first-time use of Co_3_O_4_/C-10/GCE nanocomposite for the highly sensitive detection of PLB. TEM images revealed that Co_3_O_4_/C-10 has a more homogeneous structure and higher conductivity compared to that of Co_3_O_4_/C-20, leading to the modification of GCE with Co_3_O_4_/C-10 for PLB detection. We have successfully developed a precise electrochemical sensor using Co_3_O_4_/C-10/GCE for detecting PLB. This represents the first attempt to produce a nanosensor for detecting PLB in urine or drugs using Co_3_O_4_/C-10/GCE, which has made significant progress in this field. The analysis demonstrated excellent reproducibility, repeatability, and with specificity against various interferences. The developed nanosensor operated within a concentration range of 1.0–5.0 μM and had a limit of detection (LOD) of 0.122 μM. Recovery values ranged from 99.3% to 101.3%. The suitability and practical potential of the nanosensor for quality control and research applications are evident. This review has validated the method. Since our developed sensor does not use hazardous reagents or solvents that could pose risks to human health or the environment, our method can be considered a green chemistry approach. Moreover, our approach represents a significant advancement in providing effective drugs for medical tests and their precise detection. It offers an economically feasible alternative to existing analytical methods and is easily applicable. We also anticipate the use of this developed sensor as an alternative in routine and clinical analyses of anticancer drugs in the CDK4/6 inhibitor group. This study reports the novelty of the Co_3_O_4_/C-10/GCE method, which provides various advantages in analytical laboratories, enhanced cost-effectiveness, and eliminated sample preparation techniques, thus offering greater specificity. Such innovative developments will bring significant changes to future pharmaceutical analyses.

## Supporting Information Available

TEM images and SEM-EDS mapping of Co_3_O_4_/C-10 and Co_3_O_4_/C-20, the content of elements in Co_3_O_4_/C-10 according to XPS analyses, XRD patterns, N_2_ adsorption isotherms, and SEM images of the ZIF-12, CV curves at different scan speeds on bare GCE and Co_3_O_4_/C-10/GCE, etc. (PDF)
