# Supplementary material for: Superior Electrochemical Sensor Application of Co3O4/C Heterostructure in Rapid Analysis of Anticancer Drug Palbociclib in Pharmaceutical Formulations and Biological Fluids
Source: Langmuir. 2024 Sep 28;40(40):21139–51. doi: 10.1021/acs.langmuir.4c02551 (PMC11465771; doi:10.1021/acs.langmuir.4c02551)
Supplement: Supplementary file 1 — la4c02551_si_001.pdf [file la4c02551_si_001.pdf]

Supporting Information

Superior Electrochemical Sensor Application of Co_3O_4/C Heterostructure in Rapid Analysis of Anticancer Drug Palbociclib in Pharmaceutical Formulations and Biological Fluids

Ozgul Vural[1,2]*, Nesrin Buğday[3], Asena Ayşe Genc[1,2], Nevin Erk[1], Ozgur Duygulu[4], Sedat Yaşar[3]*

[1] Ankara University, Faculty of Pharmacy, Department of Analytical Chemistry, 06560 Ankara,

Turkey , vuralo@ankara.edu.tr , asenaaysegenc@gmail.com ,erk@pharmacy.ankara.edu.tr,

[2] Ankara University, The Graduate School of the Health Sciences, 06110 Ankara, Turkey

[3] İnönü Üniversity, Faculty of Science and Art, Department of Chemistry, 44280, Malatya, Turkey, nbugday7@gmail.com, sedat.yasar@inonu.edu.tr

[4] TUBITAK Marmara Research Center, Materials Technologies, 41470 Gebze, Kocaeli, Turkey, ozgur.duygulu@tubitak.gov.tr

Email corresponding authors: sedat.yasar@inonu.edu.tr; vuralo@ankara.edu.tr

Table of Contents

1. Materials, Reagents and Instrumentation..S3

Figure S1. TEM images of Co_3O_4/C-10 (a) and Co_3O_4/C-20 (b) **.........................S4**

Figure S2. SEM-EDS mapping of C, O, and Co of Co_3O_4/C-10 (a) and Co_3O_4/C-20(b) **...S5**

Table S1. The content of elements in **Co_3O_4/C-10** according to XPS analyse**............S5**

Figure S3. XRD patterns (a), N_2 adsorption isotherms (b) and SEM images (c) of the ZIF-12.......... ....**S6**

Figure S4. The recorded CV curves (A) ,and the relationship between Ipa vs v1/2 (B) on the bare GCE at various scan rate in 5.0 mM [Fe $(CN)_6]^{3-/4-}$ containing 0.1 M KCl. **S6**

Figure S5. Using Co_3O_4/C-10/GCE, the recorded CV curves at different scan speeds with 5.0 mM [Fe $(CN)_6]^{3-/4-}$ containing 0.1 M KCl. **S7**

Figure S6. Influence of supporting electrolyte (A), the concentration (B), and the amount (C) of Co_3O_4/C-10/GCE composite on the oxidation peak currents of 0.1 mM PLB. **S8**

Figure S7. Tafel plot for 0.1mM PLB with scan rates of 100 mV s^{-1} at the surface of Co_3O_4/C-10/GCE. **S9**

Figure S8. The 0.01 mM PLB at Co_3O_4/C-10/GCE in B-R buffer (pH 2.0) was tested for repeatability (A), reproducibility (B), Selectivity (C) and Stability (D). **S10**

Table S2. Effect of different interferents on theoxidationcurrent of PLB (1.0 μM) at Co_3O_4/C-10/GCE. **S11**

1. Materials, Reagents and Instrumentation

To be used in the studies, the following reagents were bought at Sigma Aldrich Co. (Germany): 99.5% glucose; 98.0% L-arginine; L-methionine; Sodium hydroxide; 99% $K_3Fe(CN)_6$; HCl; sodium acetate; ascorbic acid; 99.0% uric acid; acetic acid; potassium chloride; sodium phosphate; sodium sulfate. These were analytic grade chemicals that were used directly without further purity. The source for these reagents is Sigma Aldrich Co., and more information can be found on their website: Sigma Aldrich Co. (https://www.sigmaaldrich.com, Germany). Phosphoric, boric, and acetic acids were dissolved in highly pure water to create Britton-Robinson (B-R) buffer. The stock solution for PLB was prepared using a methanol: water at 1:1 proportion. Chemical compounds were used directly as they were of analytical purity.

XRD analysis of the prepared materials was performed outusing Rigaku Rint 2000 X-ray Diffractometer between 2 and 80 /min with a scan rate of 2/min. The specimens were investigated by JEOL JSM 6510LV scanning electron microscope (SEM) at 15 kV. The morphology at nanoscale was further observed by JEOL JEM 2100 High Resolution Transmission Electron Microscope (LaB_6 filament) operated at 200 kV and equipped with an Oxford Instruments X-Max 80T Energy Dispersive Spectrometer (EDS) system. Carbon support film coated copper TEM grids (Electron Microscopy Sciences, CF200-Cu, 200 mesh) were used. Images were taken by Gatan Model 833 Orius SC200D CCD Camera. Gatan Microscopy Suite (GMS) 2 software was used. For diffraction pattern indexing CrystBox software was used [M. Klinger. CrysTBox - Crystallographic Toolbox. Institute of Physics of the Czech Academy of Sciences, Prague, 2015. ISBN 978-80-905962-3-8. URL http://www.fzu.cz/~klinger/ crystbox.pdf]. The elemental composition and phase structure were analyzed by X-ray photoelectron spectroscopy (XPS) were recorded using a Specs-Flex XPS instrument in the range of 200-4000 eV. Benzimidazole (BIM), Cobalt acetate tetrahydrate ($(CH_3COO)_2Co.4H_2O$), sodium chloride (NaCl), potassium iodate (KIO_3) and NH_4OH (NH_3, 28–30% aqueous solution) were obtained from Sigma-Aldrich and used without any purification.

The electrochemical evaluation was conducted utilizing the METROHM-Autelab potentiostat/galvanostat apparatus (Model 128N, software version Nova 2.6.1). Ag/AgCl (3 M KCl) was used as the reference electrode, GCE and Co_3O_4/C/GCE as the working

electrodes, and a Pt wire purchased from BASi® was used as the counter electrode in a three-electrode setup. The urine sample used in the experiment was provided by a volunteer

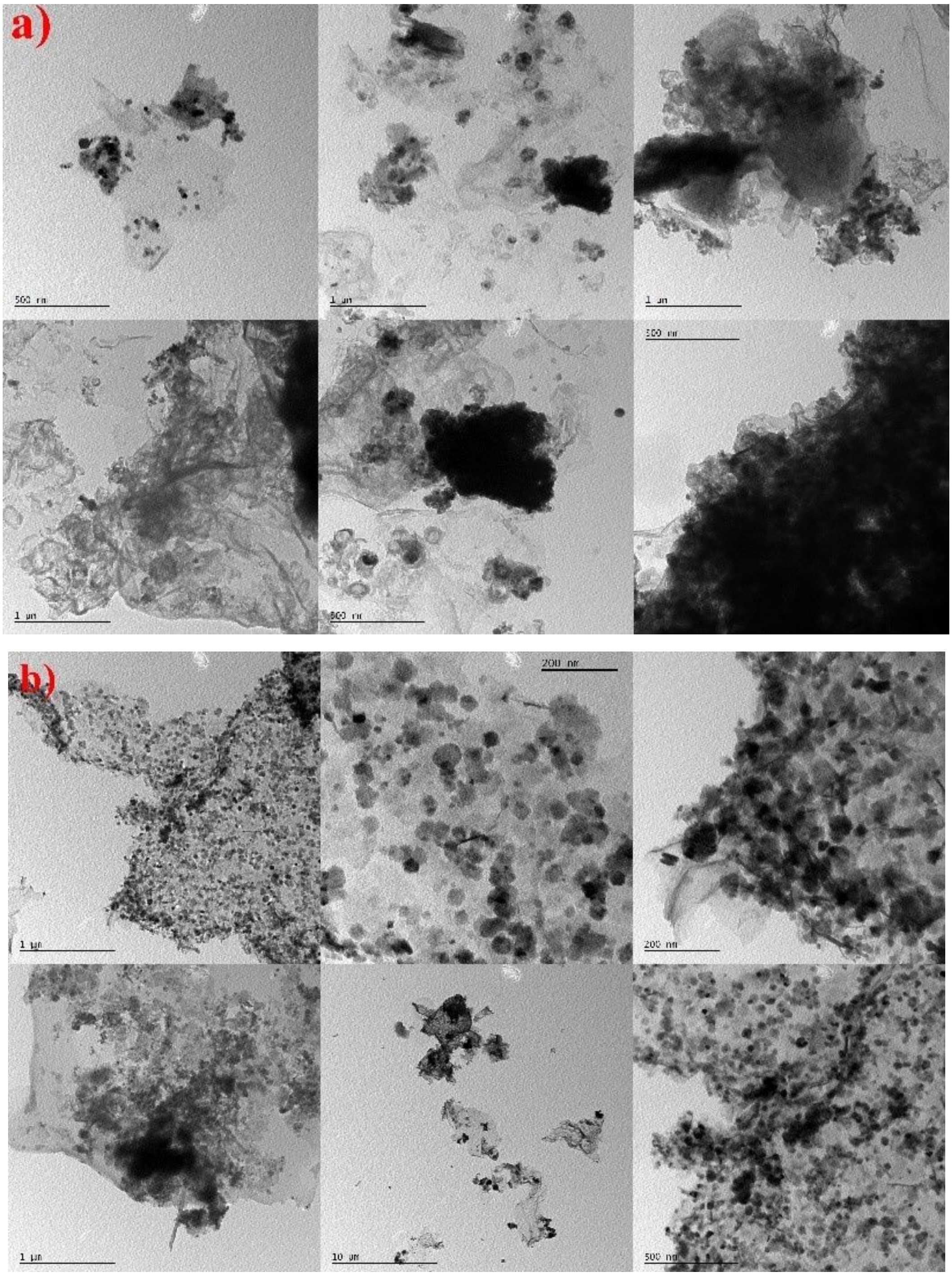

Figure S1. TEM images of Co_3O_4/C-10 (a) and Co_3O_4/C-20 (b)

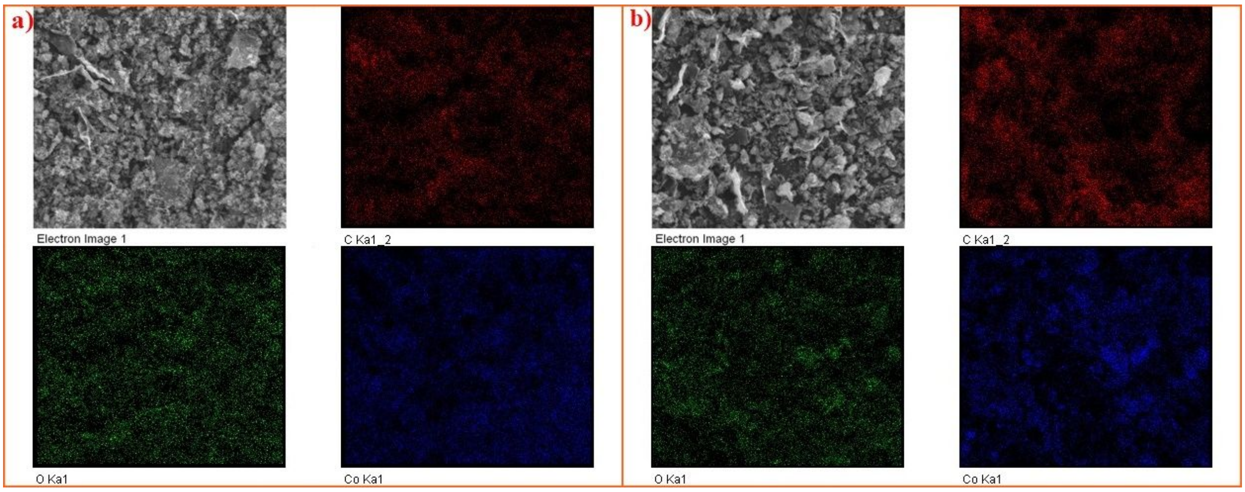

Figure S2. SEM-EDS mapping of C, O, and Co of Co_3O_4/C-10 (a) and Co_3O_4/C-20 (b)

Table S1. The content of elements in **Co_3O_4/C-10** according to XPS analyse

**XPS**	**At%**
C 1s	70.15
O 1s	18.31
Co $2p_{3/2}$	6.99
N 1s	4.54

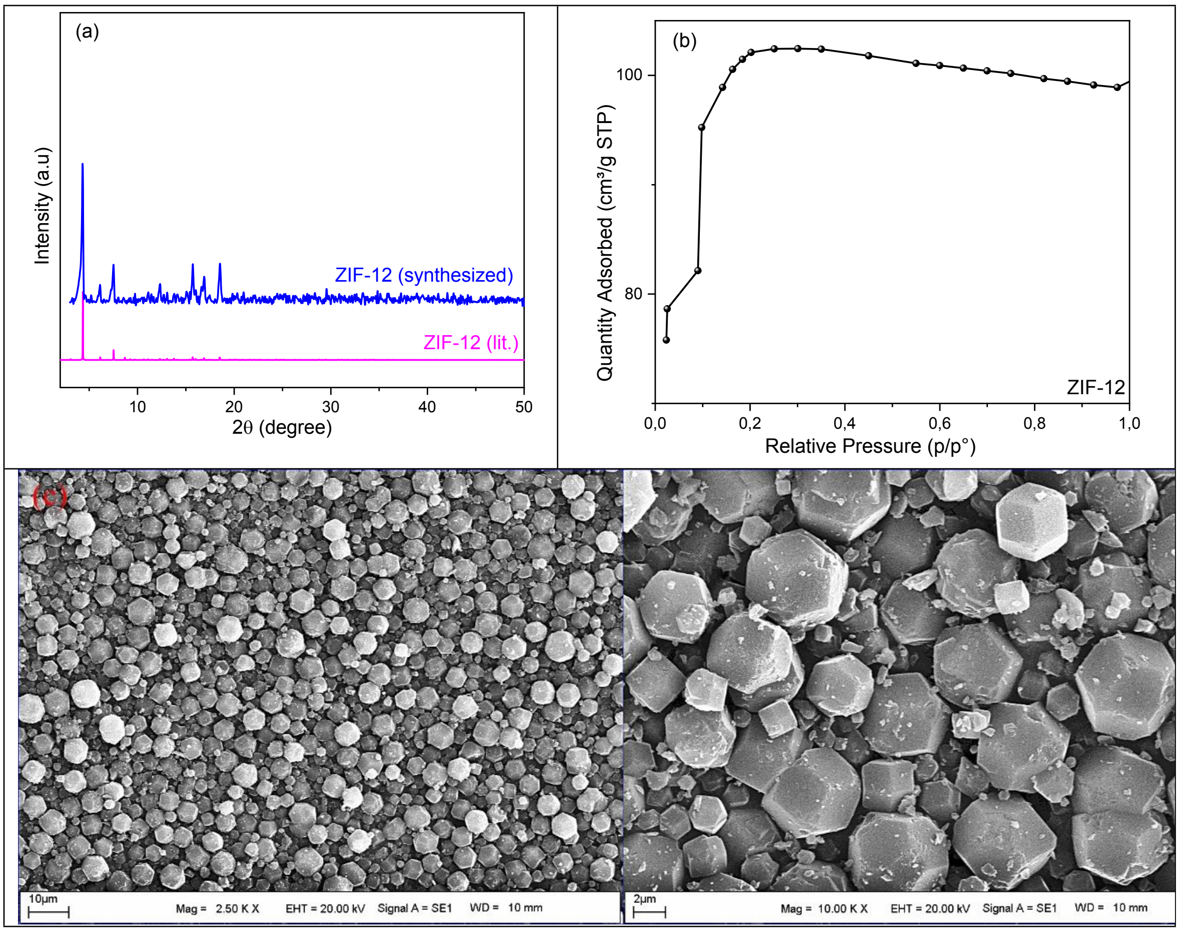

Figure S3. XRD patterns (a), N_2 adsorption isotherms (b) and SEM images (c) of the ZIF-12.

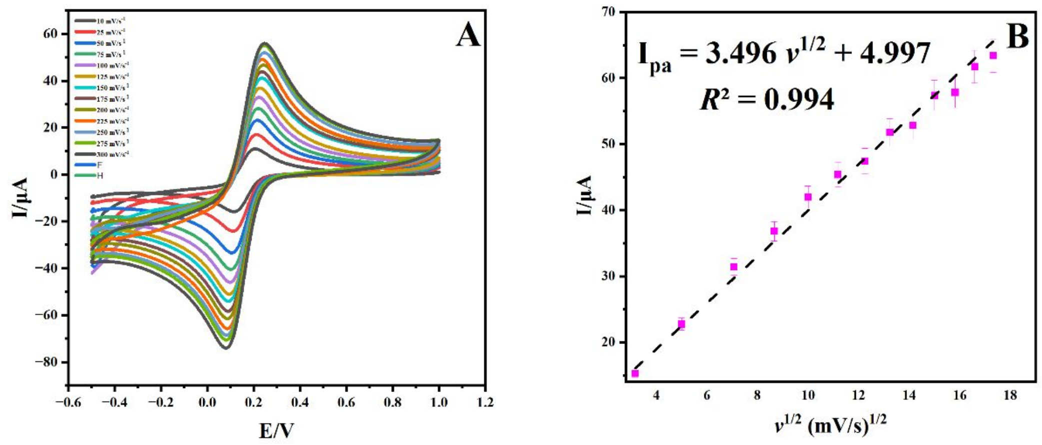

Figure S4. The recorded CV curves (A), and the relationship between Ipa vs v1/2 (B) on the bare GCE at various scan rate in 5.0 mM $[Fe\ (CN)_6]^{3-/4-}$ containing 0.1 M KCl.

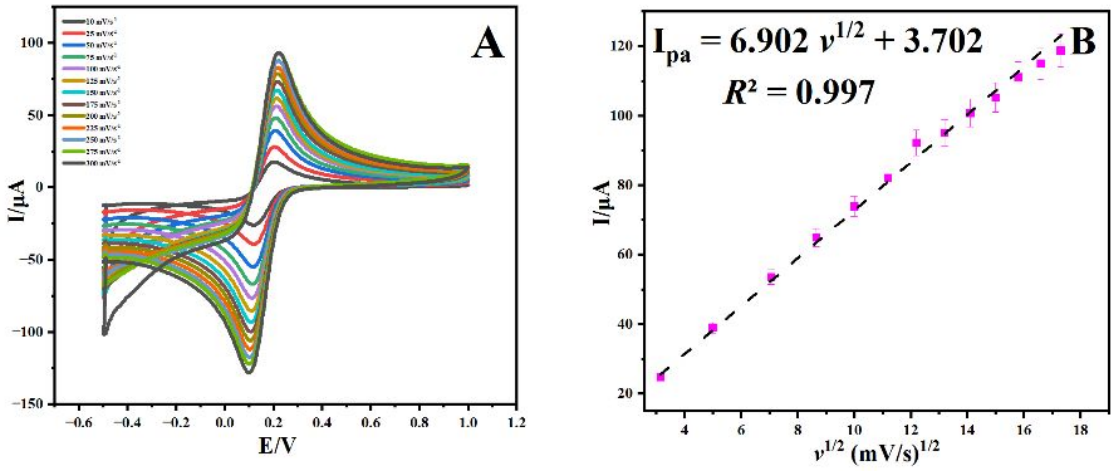

Figure S5. The recorded CV curves (A), and the relationship between Ipa vs v1/2 (B) using Co_3O_4/C-10/GCE at various scan rate in 5.0 mM [Fe $(CN)_6]^{3-/4-}$ containing 0.1 M KCl.

The Randles-Sevcik equation:

$$I = (2.69 \times 10^5)\, n^{\frac{3}{2}}\, A\, D^{\frac{1}{2}}\ v^{\frac{1}{2}}\, C_0 \qquad (S1)$$

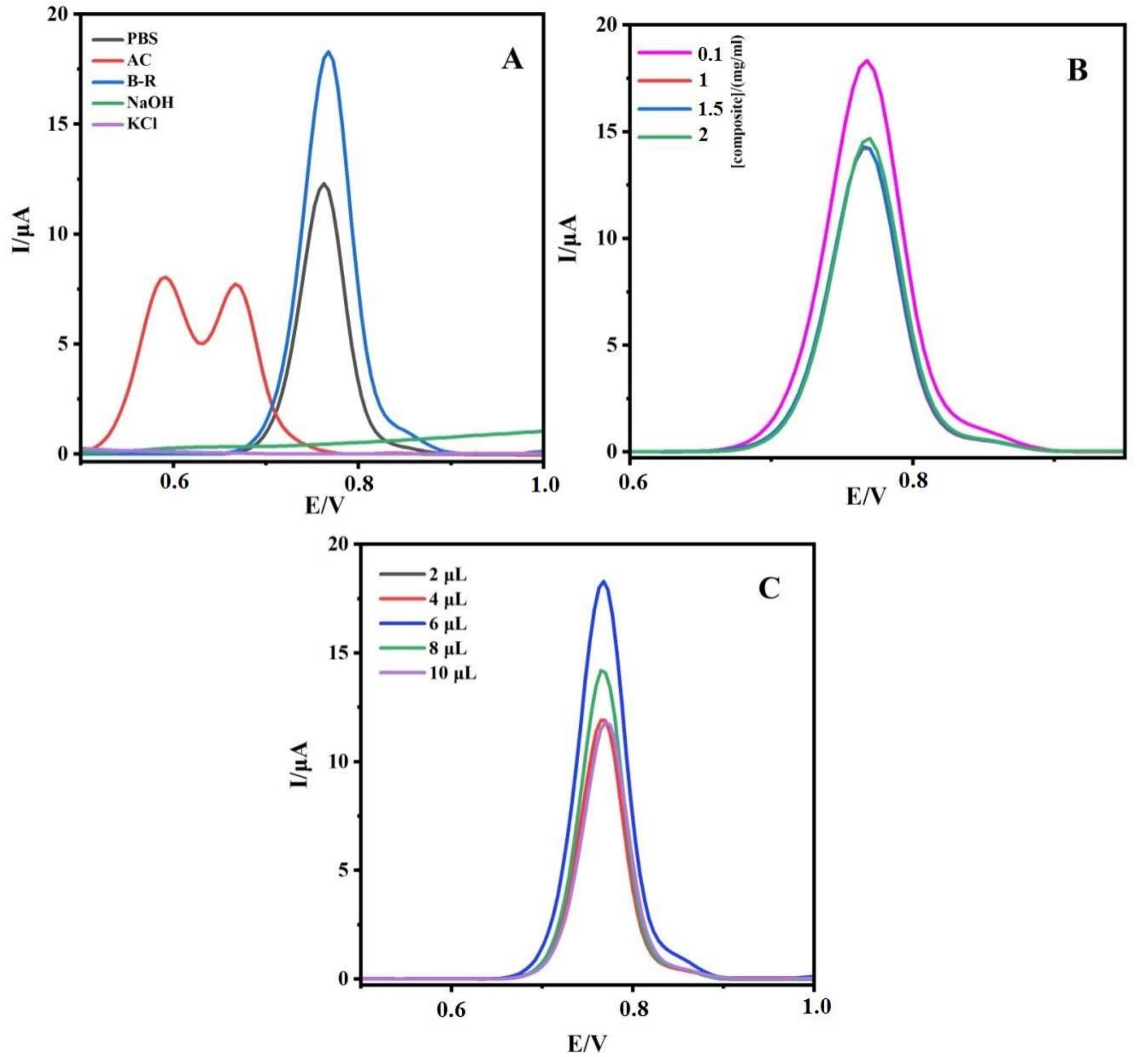

Figure S6. Influence of supporting electrolyte (A), the concentration (B), and the amount (C) of Co_3O_4/C-10/GCE composite on the oxidation peak currents of 0.1 mM PLB.

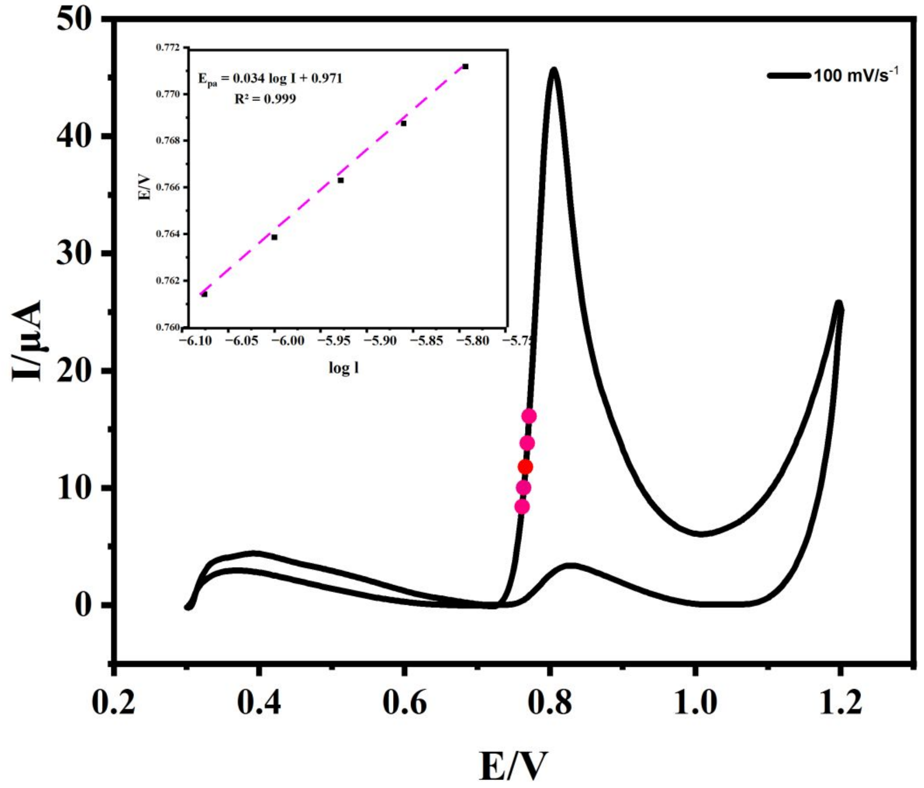

Figure S7. Tafel plot for 0.1mM PLB with scan rates of 100 mV s^{-1} at the surface of Co_3O_4/C-10/GCE.

The Tafel equation:

$$log|j_{ct}| = log j_0 + \frac{(1-\alpha)}{2.3RT}|\eta_{ct}| \qquad (S2)$$

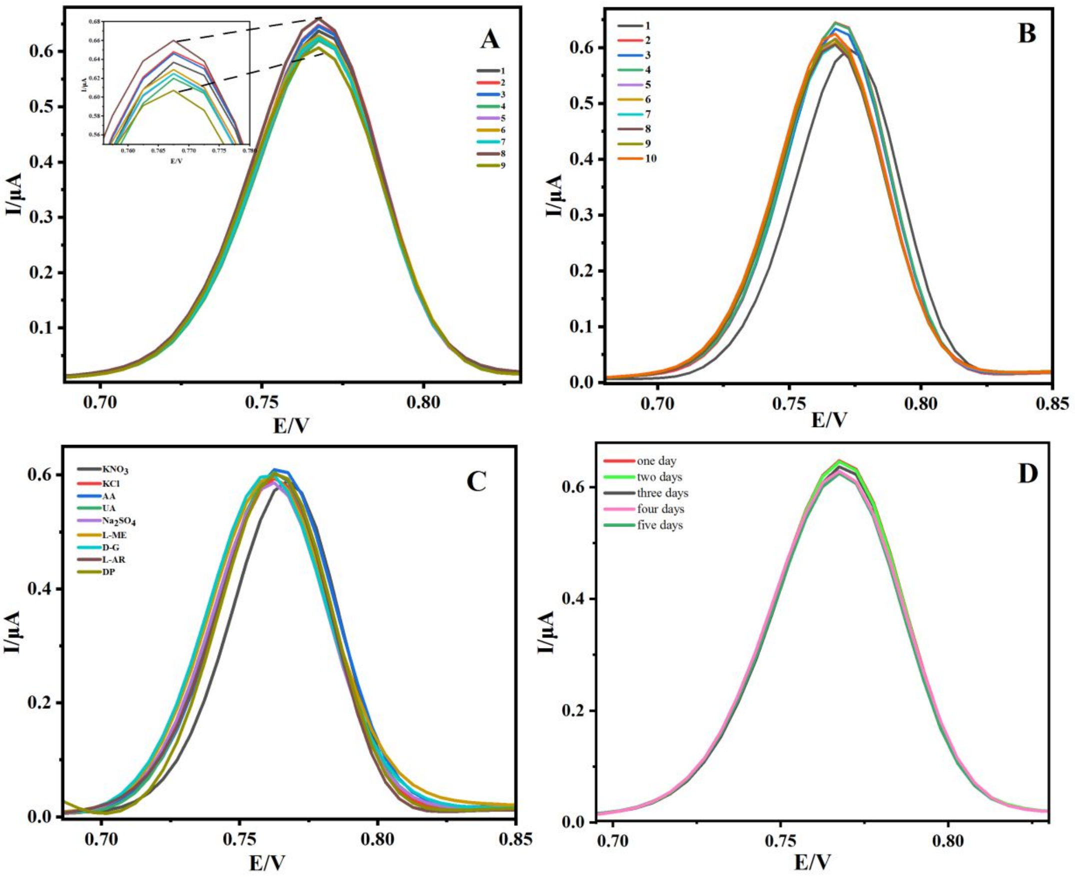

Figure S8. The 0.01 mM PLB at Co_3O_4/C-10/GCE in B-R buffer (pH 2.0) was tested for repeatability (A), reproducibility (B), Selectivity (C) and Stability (D).

Table S2. Effect of different interferents on theoxidationcurrent of PLB (1.0 μM) at Co_3O_4/C-10/GCE.

Interferent	PLB:Interferent	RSD(%)	Signal Change (%)
**PLB**	1:100	0.10	-
**KCl**	1:1000	0.29	0.40
**DOPA**	1:100	1.02	-0.91
**AA**	1:1000	0.95	-0.85
**L-M**	1:1000	0.84	0.94
**UA**	1:1000	0.78	-0.67
**KNO_3**	1:1000	0.51	0.61
**Na_2SO_4**	1:1000	2.00	2.10
**D-G**	1:1000	0.17	-0.07
**L-A**	1:1000	0.62	-0.51
